# Transforming Heart Failure Management: The Power of Strain Imaging, 3D Imaging, and Vortex Analysis in Echocardiography

**DOI:** 10.3390/jcm13195759

**Published:** 2024-09-27

**Authors:** Domenico Galzerano, Maria Teresa Savo, Biagio Castaldi, Naji Kholaif, Feras Khaliel, Alice Pozza, Saif Aljheish, Irene Cattapan, Marika Martini, Eleonora Lassandro, Gabriele Cordoni, Donatella Tansella, Dan Alexandru Cozac, Bandar Alamro, Giovanni Di Salvo

**Affiliations:** 1 College of Medicine, Alfaisal University, Riyadh 11533, Saudi Arabia; dr.kholaif@gmail.com (N.K.); b.alamro@hotmail.com (B.A.); 2 Heart Centre, King Faisal Specialist Hospital & Research Centre, Riyadh 11564, Saudi Arabia; fekhaliel@ksfhrc.edu.sa (F.K.); saif.aljheish@gmail.com (S.A.); 3 Cardiology Unit, Cardio-Thoraco-Vascular and Public Health Department, Padova University Hospital, 35121 Padova, Italy; mariateresa.savo@studenti.unipd.it (M.T.S.); marika.martini.1@phd.unipd.it (M.M.); eleonora.lassandro@studenti.unipd.it (E.L.); gabriele.cordoni@studenti.unipd.it (G.C.); donatella.tansella@studenti.unipd.it (D.T.); 4 Division of Pediatric Cardiology, Department for Women’s and Children’s Health, University of Padua, 35128 Padua, Italy; biagio.castaldi@unipd.it (B.C.); irene.cattapan@phd.unipd.it (I.C.); giovanni.disalvo@unipd.it (G.D.S.); 5 Emergency Institute for Cardiovascular Diseases and Transplantation of Targu Mures, 540136 Targu Mures, Romania; dan.alexandru03@yahoo.com

**Keywords:** heart failure, advanced cardiac imaging, three-dimensional echocardiography, myocardial strain imaging, vortex dynamics

## Abstract

Heart failure (HF) remains a critical global health challenge, necessitating advancements in diagnostic and therapeutic strategies. This review explores the evolution of imaging technologies and their impact on HF management, focusing on three-dimensional echocardiography (3DE), myocardial strain imaging, and vortex dynamics imaging. Three-dimensional echocardiography enhances traditional echocardiography by providing more accurate assessments of cardiac structures, while myocardial strain imaging offers the early detection of subclinical myocardial dysfunction, crucial in conditions such as chemotherapy-induced cardiotoxicity and ischemic heart disease. Vortex dynamics imaging, a novel technique, provides insights into intracardiac flow patterns, aiding in the evaluation of left ventricular function, valve diseases, and congenital heart anomalies. The integration of these advanced imaging modalities into clinical practice facilitates personalized treatment strategies, enabling the earlier diagnosis and more precise monitoring of disease progression. The ongoing refinement of these imaging techniques holds promise for improving patient outcomes and advancing the field of precision medicine in HF care.

## 1. Introduction

Heart failure (HF) remains a critical global health issue, affecting millions of patients worldwide. The evolution of imaging technologies has significantly impacted HF management, providing clinicians with advanced tools to assess cardiac function and tailor treatment strategies. Two-dimensional echocardiography (2DE) uses sound waves to create images of the heart. It is widely available and provides essential information about the cardiac structures and function. However, it has limitations in detecting subtle myocardial changes and complex flow dynamics [1,2]. Despite these limitations, 2DE remains a cornerstone for the initial cardiac assessment [3,4]. Cardiac magnetic resonance imaging (MRI) utilizes magnetic fields to produce detailed images of the heart. It offers excellent structural and tissue characterizations, making it invaluable for diagnosing various cardiac conditions. However, the cost and limited availability can restrict its use [1,2]. Traditional imaging techniques have been complemented by innovations such as three-dimensional echocardiography (3DE), myocardial strain imaging, and vortex analysis, which offer deeper insights into cardiac function and dysfunction (Table 1) [5,6]. This review explores these advancements and their implications for HF management.

## 2. Three-Dimensional Echocardiography

Three-dimensional echocardiography has expanded the capabilities of traditional 2DE by providing a presentation of the cardiac structure from any spatial point of view. To create larger volumetric data, multiple beat 3DE acquisition acquires narrow volumes of information over several heartbeats that are then stitched together. This compensates for the poor temporal resolution of single beat full volumetric real-time 3DE acquisition but has the disadvantage of having a stitch artifact. The presence of respiratory motion or irregular cardiac rhythms can create artifacts [7]. 

### 2.1. Clinical Applications

**Figure 1:**
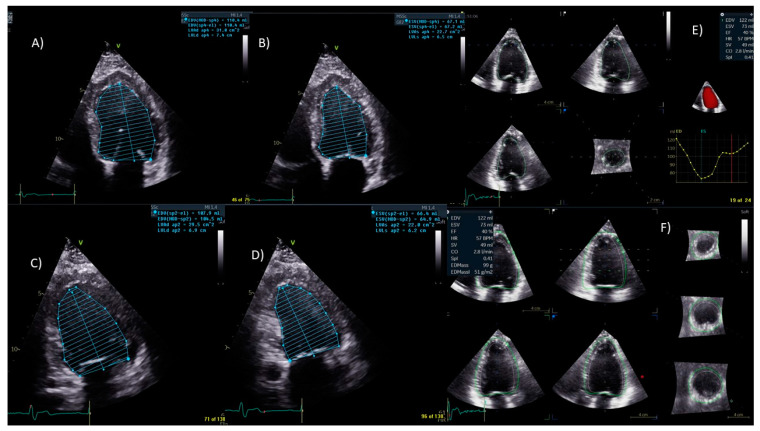
**Two-dimensional and three-dimensional LVEF valuation.** In presence ofminimal differences in the ejection fraction measurement, the indications for medical therapy or possible resynchronization therapy can shift. In this case, the volumes were measured both with the 2D and 3D Simpson’s methods of disks (MODs). Panels (**A**,**B**): the left ventricular (LV) ejection fraction is 38%, measured with the MODs. The volumes in diastole and systole are 110 mL and 67 mL. Panels (**C**,**D**): the MODs in the two-chamber view. In diastole and systole, the volumes are, respectively, 107 mL and 66 mL. Panels (**E**,**F**): LV ejection fraction of 40% with volumes of 73 mL and 122 mL. Although the ejection fraction was similar, there was a difference in the volumes, underscoring a likely systematic error in measuring the volumes in two-dimensional method.

1. **Left ventricular function:** This technology allows for a more accurate evaluation of the left ventricle (LV) function, avoiding geometric assumptions regarding the LV shape. It provides faster, more accurate, and reproducible measurements of ventricular volumes, compared to traditional 2DE (Figure 1).

**Figure 2:**
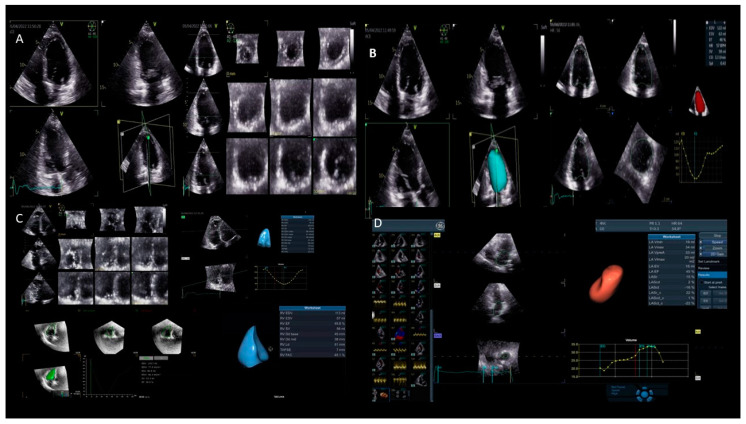
**Possible applications of 3DE.** Panel (**A**) shows a 3DE image “full volume” for the left ventricular volume; the orthogonal views confirm wall motion abnormalities in any given segment. Panel (**B**) shows the 3D left ventricular volume and function. Panel (**C**) shows the 3D right ventricular volume and function. Panel (**D**) shows the 3D left atrium volume and function.

The accuracy of 3DE is comparable to a cardiac MRI; however, the variability in the results may be greater due to differences in the image quality and operator expertise. Two primary approaches can be used: the first one utilizes a “full-volume” data set to generate standard 2DE images, with the careful optimization of the cut planes to ensure they are aligned “on axis” (Figure 2). This method is particularly effective for assessing the segmental wall motion and for tracing the LV borders to calculate the volumes. In segmental imaging, obtaining orthogonal views offers the advantage of confirming wall motion abnormalities in any given segment. This is important in conditions such as HF, cardiomyopathies, and cardio-oncology, where the precise quantification of the cardiac function is essential for guiding treatment decisions and monitoring disease progression. The second approach involves the visualization of rendered 3DE images, which provide a comprehensive impression of cardiac structures, such as the LV mass [7].

**Figure 3:**
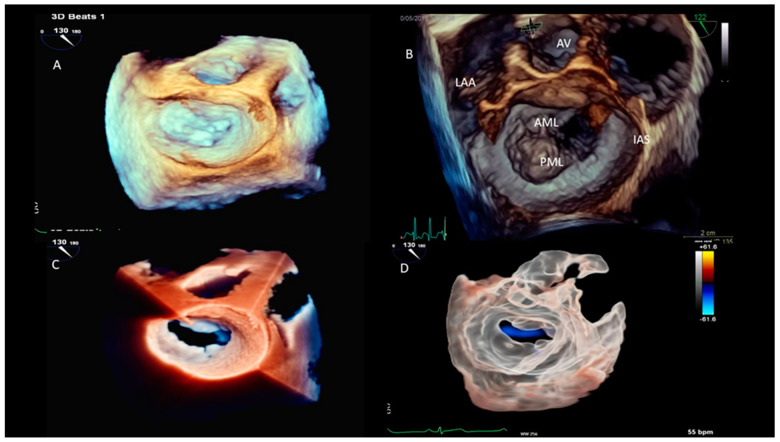
**Three-dimensional image of mitral valve surgical atrial view.** Panel (**A**) shows normal mitral valve with 3D echocardiography evaluation; panel (**B**) showsprolapse of mitral valve scallop P2; panel (**C**) shows normal mitral valve with TrueVue view modality; and panel (**D**) GlassVue view of normal mitral valve (LAA, left atrial appendage; AV, aortic valve; IAS, interatrial septum; AML, anterior mitral leaflet; and PML, posterior mitral leaflet).

2. **LV desynchrony:** For this assessment, the segmental LV volumes are tracked throughout the cardiac cycle. This temporal analysis allows for the identification of differences in the timing of each segment reaching its minimal volume, which corresponds to the maximal contraction. Under normal physiological conditions, all the LV segments reach their minimal volume simultaneously during ventricular systole. However, in the presence of dyssynchrony, there is a temporal dispersion, with diseased segments achieving the minimal volume later in systole. The systolic dyssynchrony index (SDI) quantifies dyssynchrony by calculating the standard deviation of the times to the regional minimal volume across all the segments. Studies have demonstrated that the SDI is a strong predictor of cardiac resynchronization therapy (CRT) response, with significant predictive power observed at 48 h [8], as well as at 6-month and 1-year follow-ups [9]. Additionally, the importance of the optimal LV pacing lead placement has been highlighted in studies using 3DE. Patients with pacing leads positioned at the site of the maximal mechanical delay experienced significantly greater improvements in the LV function, reverse remodeling, and peak oxygen consumption compared to those with leads placed distal to the optimal site [10].
3. **Valve assessment:** Three-dimensional echocardiography offers detailed visualization of the heart valves, which is crucial for diagnosing and planning surgical interventions in patients with valve diseases, such as stenosis or regurgitation (Figure 3). The ability to visualize the valves in three dimensions allows clinicians to measure the exact size and shape of the valve orifice, the extent of leaflet prolapse, and the severity of regurgitation or stenosis. This detailed assessment helps in selecting the most appropriate treatment strategy, whether it be surgical repair, valve replacement, or percutaneous interventions.
4. **Interventional procedure and cardiac surgery:** It is used for intraoperative monitoring during complex cardiac surgeries, providing real-time visual guidance that can improve surgical outcomes. In procedures such as transcatheter aortic and mitral valve replacements, mitral valve repairs, or the closure of atrial septal defects, real-time 3DE imaging provides invaluable guidance. It allows for the accurate positioning of catheters and devices, ensuring optimal procedural outcomes and reducing the risk of complications. The ability to visualize the heart and the devices in three dimensions enhances the precision of these interventions and improves patient safety.
5. **Assessment of atrial function:** It allows for the detailed evaluation of the atrial size, shape, and function, which is important in conditions such as atrial fibrillation and atrial septal defects. The ability to visualize the atria in three dimensions provides a better understanding of their pathophysiology and helps in planning interventions such as catheter ablation or the surgical closure of defects [11,12,13].

### 2.2. Limitations

One of the main limitations of 3D echocardiography, in addition to being an operator-dependent technique, is the trade-off between spatial and temporal resolutions. Achieving a higher spatial resolution requires the acquisition of a greater number of scan lines per volume, which in turn increases the time required for the image acquisition and processing, ultimately reducing the temporal resolution. To maintain an adequate temporal resolution, it is often necessary to compromise by acquiring smaller volumes. Additionally, the presence of arrhythmias poses another significant challenge, because they complicate reliable recording R-R intervals. In such cases, increasing the number of cardiac cycles while reducing the acquisition volume can serve as a feasible compromise to enhance the temporal resolution. Lastly, optimal 3D imaging relies on high-quality 2D images, with efforts to minimize respiratory artifacts during acquisition. In 3D color Doppler imaging, it can be difficult to strike an optimal balance between spatial and temporal resolutions with existing technology. Opting for a smaller acquisition volume can be a beneficial strategy to overcome this limitation.

## 3. Myocardial Strain Imaging

Strain imaging is an advanced echocardiographic technique that has greatly improved the assessment of myocardial function. By measuring the deformation of the cardiac muscle during the cardiac cycle, strain imaging provides detailed insights into the myocardial mechanics (Table 2) that traditional measures of cardiac function, such as the ejection fraction, cannot provide [11,14]. In clinical practice, myocardial deformation is typically described using three orthogonal components: longitudinal, radial, and circumferential. The circumferential–longitudinal LV shear strain is expressed as rotation, twist, or torsion, though, like other shear strain components, it is rarely applied in clinical settings. Myocardial fibers contribute to both longitudinal and circumferential shortening, depending on their orientation. Radial deformation results from fiber shortening across all layers, enhanced by the thickening and inward movement of the myocardium. Due to the intricate architecture of the LV, a fiber shortening of just 15% can translate into a 60% reduction in the LV volume. This structure also means that regional pathology often affects all three strain components, allowing clinicians to select the most reliable component for measurement. Longitudinal strain is the most commonly used deformation measure, as it is relatively uniform along the LV wall and enables the comprehensive assessment of the entire LV from just three apical views, making it simple and reliable to use [15]. Global strain, most often assessed as global longitudinal strain (GLS), evaluates only one of the deformation components. Studies have demonstrated that strain imaging is more effective at identifying subtle systolic dysfunction than the left ventricular ejection fraction (LVEF). This is likely because the heart can compensate for early longitudinal dysfunction by increasing other strain components, allowing the LV to maintain a normal ejection fraction despite early impairment [16].

### 3.1. Clinical Applications

**Figure 4:**
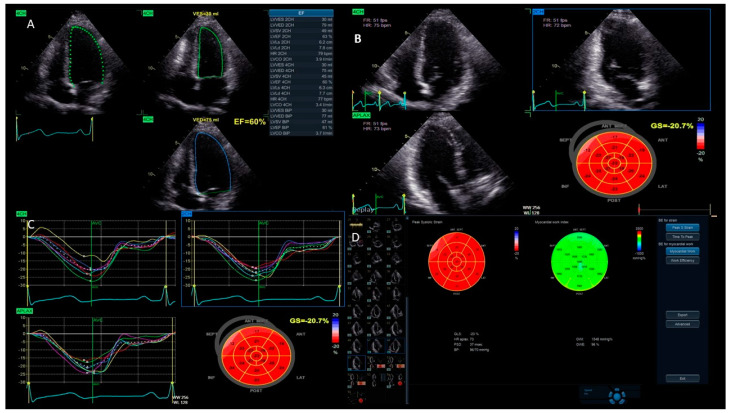
**Myocardial strain valuation**. Panel (**A**) shows the auto ejection fraction calculation with a good quality apical acoustic window of the endocardial edges with artificial intelligence technology that allows semi-automatic recognition; in this image, the LVEF = 60%. Panels (**B**,**C**) show a 2D left ventricular echocardiography with speckle tracking with a normal global longitudinal strain (GLS) in the range of −21.7%. Panel (**D**) shows a normal global longitudinal strain (GLS) of −20% and a normal myocardial work index of 1548 mm Hg%.

1. **Left ventricular function:** One of the main uses of strain imaging is to detect early subclinical myocardial dysfunction. For instance, in patients undergoing chemotherapy, strain imaging can detect early signs of heart damage before a significant reduction in the LVEF occurs. This allows for prompt intervention and adjustments to the cancer treatment to prevent further harm to the myocardium [17,18]. The current definitions of cancer therapy-related cardiac dysfunction primarily rely on a reduction in the LVEF and/or a relative decrease in the GLS beyond a specific threshold [19]. As a result, baseline cardiac assessments are recommended for all patients prior to initiating cardiotoxic cancer treatments. GLS assessment using speckle tracking, particularly from three apical views, is strongly advised at the baseline, especially for patients at a moderate-to-high risk. It is important to acknowledge that strain measurements may vary between different vendors. Therefore, to ensure consistency, serial GLS evaluations for each patient should be conducted using the same equipment and software. A median GLS reduction of 13.6% has been identified as a predictor of future LVEF decline, with an upper limit of 15% recommended as the threshold for GLS reduction during cancer therapy to enhance specificity [19,20]. These measurements help stratify the risk of cancer treatment-related cardiovascular toxicity and identify significant changes during therapy. Notably, a normal LVEF does not exclude the presence of cancer treatment-related cardiac dysfunction; GLS, instead, can reliably detect early systolic impairment. For instance, Muckiene et al. demonstrated that a reduction in GLS is significantly linked to early anthracycline-induced cardiotoxicity in patients undergoing anthracycline-based chemotherapy. This finding suggests that GLS could potentially serve as a predictor for any subsequent declines in the LVEF associated with this chemotherapy regimen [21]. In the assessment of ischemic heart disease, strain imaging provides valuable information about regional myocardial function [16]. During an ischemic event, specific areas of the myocardium may show reduced strain, indicating impaired contractility. This technique can help identify a viable but hibernating myocardium, which can benefit from revascularization procedures. Strain imaging is also useful in evaluating the effectiveness of reperfusion therapies following acute myocardial infarction by assessing the recovery of myocardial function in the affected regions [22,23,24]. Furthermore, the management of HF patients benefits significantly from strain imaging. It offers a more sensitive measure of myocardial function compared to traditional echocardiographic parameters (Figure 4). GLS has been shown to correlate better with outcomes in heart failure patients, providing prognostic information that aids in clinical decision-making. In patients with HF with mildly reduced ejection fraction (HFmrEF), strain imaging can uncover subtle myocardial dysfunction that is often missed by conventional measures. Chang et al. demonstrated that in patients with HFmrEF, a LV GLS cut-off value of −11% effectively differentiated the subsequent risk of cardiovascular death [25]. This enhances the understanding and management of this complex condition [26].
2. **Cardiomyopathies:** In hypertrophic cardiomyopathy (HCM), strain imaging can identify areas of abnormal myocardial mechanics that are indicative of the disease [27,28]. Reduced strain in the thickened segments of the LV, usually correlated with the extent of late gadolinium enhancement in a cardiac MRI, can signal the presence of fibrosis and assist in assessing the risk of sudden cardiac death [29]. Similarly, in dilated cardiomyopathy (DCM), strain imaging allows for a detailed evaluation of the global and regional myocardial function, aiding in the monitoring of disease progression and response to therapy [30]. Moreover, the relative apical sparing of the GLS ratio (the average of the apical longitudinal strain/the average of the combined mid and basal longitudinal strain > 1) is typically presented in cardiac amyloidosis, both associated with light chain and transthyretin deposits [31]. A reduced longitudinal strain with an apical sparing pattern is therefore considered a typical red flag disease [32].
3. **LV dyssynchrony:** This technique evaluates the mechanical function of various segments of the LV to identify patients who are suitable for CRT and to monitor their response to the therapy [33,34]. Although cardiac imaging has not yet been proven to be effective for selecting candidates for CRT, there is emerging evidence supporting the use of strain imaging to identify the optimal placement of the pacing lead on the LV free wall [35]. Several studies have shown that positioning the lead in the area of the latest mechanical activation leads to better clinical outcomes [36]. Additionally, it is crucial to avoid placing the lateral lead over regions of transmural scarring. A peak radial strain value of less than 10% has been suggested as a marker for identifying the scar tissue [37].
4. **Valve assessment:** Strain imaging provides additional insight into the effect of valvular lesions on myocardial function. For example, in aortic stenosis, strain imaging can detect early myocardial dysfunction before the onset of obvious HF symptoms, helping to determine the optimal timing for surgical intervention [38,39]. Likewise, in mitral regurgitation, it assists in evaluating the compensatory mechanisms and identifying the point at which myocardial function starts to deteriorate, thereby aiding in the decision-making process for valve repair or replacement [40]. Characteristically, the longitudinal strain impairment detected in individuals with mitral valve prolapse is more regional than global, with a distinct involvement of the left ventricular basal inferolateral segments and a relative sparing of the apical region [41].
5. **Congenital heart disease:** Strain imaging is being used more and more in this field. It provides detailed functional assessments that are crucial for managing complex congenital anomalies. In patients with repaired congenital heart defects, strain imaging can monitor long-term myocardial function and detect early signs of dysfunction that may require further intervention [42]. A recent meta-analysis demonstrated that myocardial deformation parameters can be used for risk stratification in congenital heart disease (CHD) follow-ups, with an added clinical value over conventional echocardiography [43]. Indeed, in CHD, the anatomy of the ventricles is frequently distorted by the congenital abnormalities, the different surgeries, and the percutaneous procedures, with abnormal loading conditions related to the disease and surgical sequels, as well as residual lesions. In these conditions, the use of parameters that are independent by geometrical assumption, less affected by loading conditions, and not influenced by tethering provides obvious advantages over any geometric- or volumetric-based functional parameter [44]. Single ventricle strain was predictive of outcomes in hypoplastic left heart syndrome during the interstage period [45].

In Tetralogy of Fallot patients, the use of speckle tracking to assess longitudinal strain in the meta-analysis showed both RV global longitudinal strain and LV longitudinal strain to be prognostic of MACEs, independent of other conventional parameters, including the QRS duration and RV ejection fraction measured by an MRI [43]. This finding is particularly relevant, because the current volumetric cut-offs for pulmonary valve replacements are not well established [46]. Thus, biventricular strain should be included in the current risk stratification criteria, to better identify the proper timing for pulmonary valve implantations. In adult patients with repaired coarctation of the aorta (CoA), myocardial deformation properties of the left ventricle are frequently impaired despite a normal LVEF [47]. It has been demonstrated that this reduced LV GLS has a strong association with all-cause mortality and cardiovascular mortality and provided a superior prognostic performance compared with the LVEF [48]. Of note, in CoA patients with a normal LVEF, the coexistence of a reduced LV GLS is associated with a higher risk of all-cause mortality, compared with CoA patients with a normal LVGLS and LVEF [48].

### 3.2. Limitations

In clinical practice, speckle-tracking echocardiography has several limitations as it may be suboptimal in patients with poorly defined subendocardial borders, particularly in cases with near-field artifacts. These artifacts can reduce the accuracy of endocardial visualization, leading to errors in the automated tracing of the myocardium by the software. Moreover, automated software packages designed to trace the endocardium can be prone to error and, as a result, the manual verification of automated tracings is often required, which can be time-consuming. The most critical challenge is the variation between different vendors’ software, as differences in the algorithms used for the temporal and spatial smoothing can affect the accuracy of the strain measurements. The variability of normal values, assessed through different software packages, complicates the interpretation of the results, especially when comparing the results across different populations or using different software packages.

## 4. Vortex Dynamics Imaging

Vortex imaging is an innovative echocardiographic technique that provides a detailed visualization of intracardiac blood flow patterns. Unlike conventional Doppler imaging, which focuses on linear velocity measurements, vortex imaging captures the complex, swirling blood flow within the heart chambers, offering a more comprehensive understanding of hemodynamics [49]. The presence of vortices appears to play a key role in preventing the energy loss that would occur with a chaotic distribution of flow within the cardiac chambers [50]. During diastole, two distinct vortices can be observed within the LV. The first vortex, positioned anteriorly, exhibits a clockwise rotation across the LV inflow–outflow region, while the second vortex, located posteriorly, rotates counterclockwise [50]. The geometrical properties of the vortex in the LV are quantified by several key measures. These include the vortex area, which is scaled relative to the overall LV area, and the vortex intensity, which is the integral of the vorticity within the vortex, normalized by the total vorticity of the LV. Additionally, the vortex depth, defined as the distance from the vortex center to the LV base, and the vortex length, measured along the base–apex axis, are both normalized by the total length of the LV. The energy dynamics of the vortex flow are assessed by calculating the total kinetic energy dissipation (KED), which represents the amount of kinetic energy lost through viscous friction during the cardiac cycle. The total KED is integrated across the entire LV and is typically normalized by the average kinetic energy to ensure it is not directly influenced by variations in the LV size. This allows for a more consistent comparison across different heart sizes [50].

The evaluation of intracardiac flow dynamics has traditionally been limited by the need for a phase contrast cardiac MRI or contrast echocardiography with particle imaging velocimetry [51]. These methods are impractical for widespread use in patients. In recent years, color Doppler-based ultrasound techniques such as Vector Flow Mapping (VFM) have been developed to analyze organized vortical structures in the heart more practically [52]. VFM can detect and measure the flow motion in all directions within a scan plane by applying a series of mathematical equations to the color Doppler data [53]. This technique enables the visualization and quantification of complex flow patterns, such as those in cardiac chambers, where the vortices are believed to significantly reduce energy loss and optimize cardiac function. HyperDoppler is another non-invasive advanced echocardiographic technique that enhances the visualization of intracardiac vortices, providing a detailed assessment of the complex blood flow dynamics within the heart chambers, which is crucial for optimizing the understanding of cardiac function and energy efficiency [54]. Vortices appear to contribute to atrioventricular coupling and the redirection of ventricular blood inflow toward the outflow tracts, while maintaining blood motion and preventing the potential effects of stasis [55].

### 4.1. Clinical Applications

**Figure 5:**
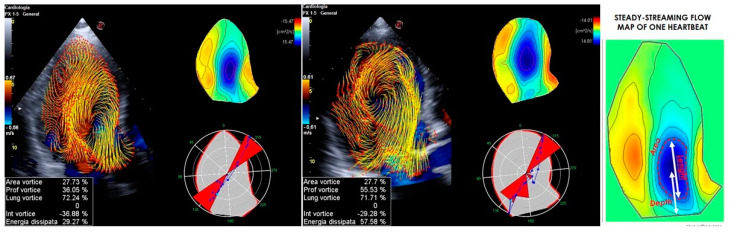
**Vortex geometric analysis.** A normal (**one the right**) versus a pathological (**on the left**) subject with aortic stenosis. The geometric values to consider are area, depth, and length. The area is identical in both the normal and pathological subjects. The depth varies between the two, with the normal subject having a smaller depth. This suggests that the aortic stenosis pushes the structure towards the tip in the normal subject. The length is the same in both subjects.

**Figure 6:**
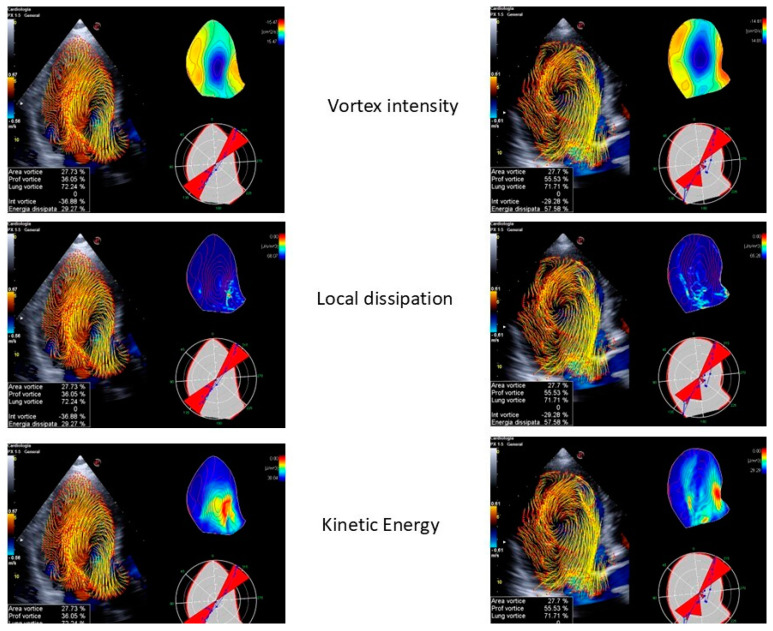
Normal vs. pathological subjects with aortic stenosis. The vortex’s energetic values to consider are the following: vortex intensity, local dissipation, and kinetic energy. The vortex intensity varies, with the normal subject exhibiting a higher intensity, compared to the pathological subject, who shows a greater local dissipation.

1. **LV function:** Vortex imaging helps clinicians visualize and measure the vortices within the LV, which are crucial for the efficient blood ejection and filling. By analyzing these flow patterns, clinicians can identify early signs of LV dysfunction that might not be obvious using standard measures. Two vortex components were consistently observed following each transmitral filling wave. The anterior vortex was analyzed due to its greater relevance in the cardiac cycle, occurring after early filling and atrial contraction. The vortex generated after early filling appears to aid LV inflow and plays a more prominent role in individuals with impaired relaxation. The vortex formed after atrial contraction seems to store kinetic energy and redirect the flow toward the outflow tract, facilitating ejection and contributing to the mitral valve closure [56]. Diastolic vortices are especially important for assessing the left atrial function and ventricular filling pressures. This is particularly valuable in conditions like heart failure with a preserved ejection fraction, where vortex dynamics can reveal underlying diastolic dysfunction [56]. Moreover, a reduced vortex formation time (VFT), a dimensionless index used to quantify the vortex development, strongly correlates with LV dysfunction and predicts adverse outcomes in patients with HF [57]. As an example, the VFT ranged between 3.3 and 5.5 in healthy subjects, but decreased to values < 2.0 in patients with dilated cardiomyopathy [58].
2. **Valve disease:** In mitral regurgitation, vortex imaging can depict the altered flow patterns caused by the regurgitant jet, helping to quantify the severity of the lesion and its effect on the LV filling. Restoring normal intracardiac LV flow patterns, as observed primarily after mitral valve replacements, may help preserve kinetic energy momentum, thereby reducing the LV workload and shear stress. A recent study revealed that intracardiac blood flow patterns are restored after mitral valve repairs, regardless of the repair technique used. In contrast, a mitral valve replacement with either biological or mechanical prostheses in non-anatomical orientations is associated with persistent alterations in the blood flow. A transcatheter edge-to-edge repair completely disrupts the LV vortices, while a transcatheter mitral valve replacement with a Tendyne valve has an effect similar to a mitral valve repair in restoring normal flow patterns [59]. Similarly, in aortic stenosis, the technique can illustrate the turbulent flow distal to the stenotic valve, offering a visual representation of the hemodynamic burden on the LV (Figure 5 and Figure 6). This information aids in the decision-making process for valve repairs or replacements by providing a more nuanced understanding of the disease’s impact on cardiac function [60,61]. Some studies have reported that aortic stenosis is associated with reduced LV filling efficiency, resulting in decreased VFT values. However, in patients with aortic stenosis and moderate aortic insufficiency, the VFT significantly increases, suggesting that the VFT may be an unreliable index of LV filling efficiency when competitive diastolic flows into the LV are present [62].
3. **Congenital heart** diseases: CHDs often involve complex intracardiac flow abnormalities that can be challenging to assess with traditional imaging techniques. Vortex imaging is particularly effective in this area, providing a detailed visualization of abnormal flow patterns, which is essential for accurate diagnosis and surgical planning. For instance, in conditions such as the Tetralogy of Fallot or the transposition of the great arteries, vortex imaging can depict the intricate flow dynamics and help in understanding the physiological consequences of the defects. In patients with transposition of the great arteries, an increased flow across the pulmonary valve secondary to a large ventricular septal defect may be responsible for a Doppler gradient at the level of the pulmonary valve, mimicking a pulmonary stenosis. In this case, the differentiation between a real valvular stenosis and a gradient secondary to volume overload is extremely important in defining the surgical timing and the type of surgery (arterial switch and ventricular septal defect closure vs. Rastelli operation). These are the kinds of situations where the traditional Doppler and color Doppler techniques demonstrate all their limitations. The use of blood speckle imaging to study flow dynamics has proven to be helpful in formulating the correct diagnosis, especially in this difficult context [63]. Post-surgical follow-ups in CHD patients also benefit from vortex imaging, as it can monitor the restoration or alteration of normal flow patterns [60].
4. **LV dyssynchrony:** Vortex imaging can assess the changes in intracavitary flow patterns before and after the CRT implantation, providing insights into the therapy’s effectiveness. Goliasch et al. utilized vortex imaging to assess the impact of an acute interruption and reactivation of the CRT. Deactivating the CRT significantly disrupted the LV filling, resulting in the reduced mitral inflow acceleration and increased total diastolic volume. This, in turn, led to the formation of an underdeveloped diastolic vortex, which impaired the transfer of kinetic energy from diastole to systole, delayed the redirection of the blood flow toward the aorta, and hindered the timely opening of the aortic valve, thereby prolonging the isovolumetric contraction period [64]. Upon the reactivation of the CRT, the LV filling improved immediately, and the total diastolic volume decreased. This restored the optimal timing of the diastolic vortex formation and shortened the isovolumetric interval [64]. By visualizing the improvement in flow efficiency and the reduction in dysfunctional vortices, clinicians can better evaluate the success of a CRT and make adjustments as needed to optimize patient outcomes.
5. **Cardiomyopathies:** In DCM, vortex imaging helps to evaluate the impact of dilated chambers on intracardiac flow and to pinpoint the regions of flow stagnation that may contribute to thrombus formation. Furthermore, vortex patterns are used to gauge the severity of HF, as fragmented or abnormal patterns are associated with increased cardiac dysfunction [65].

### 4.2. Limitations

One of the main limitations of vortex analysis is the need for specialized software, which is often expensive and not always readily available. Furthermore, this technique has not yet been fully validated in large-scale populations, highlighting the need for future studies to confirm its reliability and broader applicability.

## 5. Multimodality Imaging

### 5.1. Integrative Approach

Multimodality imaging combines various imaging techniques, such as echocardiography and a cardiac MRI, to provide a comprehensive view of the cardiac structure and function. This integrated approach enhances diagnostic accuracy and treatment planning [1]. By combining different modalities, clinicians achieve a holistic view of cardiac health, which is particularly beneficial in complex HF cases. When used together, strain imaging, 3DE, and vortex analysis provide a comprehensive assessment of cardiac function. Strain imaging identifies early myocardial dysfunction; 3D echocardiography provides more precise volumetric and structural assessments of the cardiac chambers, offering improved accuracy in evaluating heart size, shape, and function; and vortex analysis offers a deeper understanding of intraventricular flow dynamics. This multimodal approach can better define a patient’s cardiac condition, allowing for more tailored and accurate clinical decision-making. This approach also aids in monitoring the treatment by observing structural and functional changes over time (Table 3).

### 5.2. Emerging Technologies

Emerging technologies such as video-based artificial intelligence (AI) are revolutionizing HF management. Video-based AI allows for the real-time evaluation of cardiac function, improving early detection and intervention [66]. This technology provides a detailed beat-to-beat assessment, enhancing the precision of cardiac evaluations.

As an example, high frame rate imaging offers the enhanced visualization of intracardiac vortices, providing deeper insights into cardiac flow and function [67]. Blood speckle tracking may be used to understand and quantify intra- and extra-cardiac flow patterns, improving our understanding of cardiac physiology. Compared to conventional Doppler imaging, high frame rate imaging is an ultrafast technique, which generates up to thousands of frames per second and which is independent of the angle of insonation [67]. This advancement improves the analysis of vortex dynamics, contributing to a better understanding of cardiac performance and potentially leading to more effective treatments.

### 5.3. Advanced Imaging in Clinical Practice

The integration of advanced imaging technologies into clinical practice has enabled more personalized treatment strategies for HF patients. By providing detailed and accurate assessments of cardiac function, these technologies allow for tailored therapeutic approaches, improving patient outcomes. Unfortunately, vortex analysis is not yet in routine clinical practice, as it primarily remains a research tool with ongoing studies to assess its clinical relevance. However, GLS and 3DE have already been integrated into standard clinical use. GLS is particularly valuable in the assessment of cardiomyopathies, where it helps to detect the subclinical dysfunction or fibrosis and to monitor disease progression. Additionally, 3DE has proven beneficial for the more accurate evaluation of valve structures and function, offering a more comprehensive analysis compared to traditional 2DE. These advancements have improved diagnostic accuracy and patient management in daily practice. For instance, myocardial strain imaging can identify patients who would benefit most from CRT, while vortex analysis can pinpoint those at risk for diastolic dysfunction. One of the key benefits of advanced imaging is the ability to detect cardiac abnormalities early, before they manifest as clinical symptoms. Advanced imaging techniques, such as 3DE and high frame rate imaging, provide more detailed views of cardiac structures and flow dynamics, leading to enhanced diagnostic accuracy. This precision is crucial for diagnosing complex cases where traditional methods may fall short. However, difficult cases to interpret, where the risk of false positives or false negatives remains high, often require a collegial evaluation, where the echocardiographer must consult with the radiologist or the hemodynamic specialist who is about to perform or has performed a procedure.

## 6. Future Directions and Research

As technology continues to evolve, further advancements in imaging techniques are expected. These may include improvements in AI algorithms for better real-time analysis, higher resolution imaging for more detailed assessments, and the development of new modalities that can provide even deeper insights into cardiac function. The integration of advanced imaging technologies with wearable devices holds promise for the continuous monitoring of cardiac function in HF patients. This could enable real-time data collection and analysis, leading to more dynamic and responsive treatment strategies. The future of HF management lies in personalized medicine, where treatment is tailored to the individual patient’s genetic makeup, lifestyle, and specific cardiac abnormalities. Advanced imaging will play a pivotal role in this approach, providing the detailed information needed to customize treatments effectively.

## 7. Conclusions

The integration of advanced imaging technologies, such as 3DE, myocardial strain imaging, and vortex analysis, has significantly transformed HF management. These advancements provide more detailed and accurate assessments of cardiac function, facilitating earlier diagnosis, improved risk stratification, and personalized treatment strategies. As these technologies continue to evolve, they promise to further enhance HF management and patient outcomes. The ability to detect subtle myocardial changes, analyze complex flow dynamics, and integrate various imaging modalities equips clinicians with a comprehensive toolkit to address the multifaceted nature of HF. The continued refinement and development of these technologies will undoubtedly lead to significant improvements in patient care and outcomes.

## Figures and Tables

**Table 1:** Overview of traditional and advanced echocardiography techniques.

Imaging Technique	Description	Key Benefits
**2D Echocardiography**	Sound waves to create 2D images of the heart	Widely available, provides basic structural information.
**3D Echocardiography**	Provides three-dimensional images of cardiac structures	Enhanced visualization and accurate volumetric measurements.
**Myocardial Strain Imaging**	Measures myocardial deformation during the cardiac cycle	Sensitive to subtle myocardial changes, useful for the early detection of dysfunction.
**Vortex Analysis**	Analyzes swirling patterns of blood flow in the heart	Offers insights into cardiac flow dynamics and function.

MRI: magnetic resonance imaging.

**Table 2:** Key applications of myocardial strain imaging.

Application	Description	Clinical Impact
**Cardiac Function Assessment**	Evaluates myocardial deformation to identify dysfunction	Enhances the early detection of cardiac impairment
**Risk Stratification**	Uses strain metrics to predict adverse outcomes	Improves risk assessment and management strategies
**Treatment Monitoring**	Assesses changes in strain to evaluate therapy effectiveness	Provides insights into treatment responses

**Table 3:** Specific contributions of each imaging modality across various clinical scenarios, emphasizing their complementary roles in enhancing cardiac diagnosis and treatment.

Clinical Scenario	3D Echocardiography	Myocardial Strain Imaging	Vortex Imaging
**Left Ventricular Function**	Accurate evaluation of LV function, avoiding geometric assumptions. Reproducible measurements of volumes and ejection fraction. Visualization of rendered 3DE images for comprehensive cardiac structure analysis.	Detects early subclinical myocardial dysfunction, especially in conditions like chemotherapy-induced cardiotoxicity. Offers a sensitive measure of myocardial function, correlating well with outcomes in heart failure.	Visualizes and measures the intracardiac vortices crucial for efficient blood ejection and filling. Diastolic vortices assess left atrial function and ventricular filling pressures, valuable in HFpEF.
**LV Dyssynchrony**	Tracks segmental LV volumes throughout the cardiac cycle. The SDI predicts response to CRT. Optimal pacing lead placement guided by 3DE improves CRT outcomes.	Assesses desynchrony in CRT patients. Guides optimal pacing lead placement to improve outcomes.	Assesses changes in intracavitary flow patterns before and after CRT implantation. Visualizes improvement in flow efficiency post-CRT.
**Valve Assessment**	Detailed visualization of heart valves. Measures valve orifice size, leaflet prolapse, and severity of regurgitation or stenosis.	Detects early myocardial dysfunction in valvular diseases like aortic stenosis and mitral regurgitation. Helps determine timing for surgical intervention.	Depicts altered flow patterns in valve diseases like mitral regurgitation and aortic stenosis. Visualizes hemodynamic burden on the LV.
**Atrial Function**	Detailed evaluation of atrial size, shape, and function. Valuable in atrial fibrillation and atrial septal defects.	Provides global insight into cardiac function, which can include atrial contribution.	Not directly used in assessing atrial function but could offer insights into atrioventricular coupling dynamics.
**Cardiomyopathies**	Assists in the detailed assessment of LV structure and function in hypertrophic and dilated cardiomyopathy. Important for risk stratification and management.	Identifies abnormal myocardial mechanics in HCM. Assesses global and regional myocardial function in DCM, aiding in disease monitoring. Apical sparing as red flag in cardiac amyloidosis.	Evaluates the impact of dilated chambers on flow in DCM.
**Congenital Heart Diseases**	Provides comprehensive views essential for surgical planning. Monitors structural changes post-repair.	Monitors myocardial function in repaired congenital heart defects. Detects early signs of dysfunction post-surgery.	Visualizes complex flow abnormalities in congenital heart disease. Monitors restoration of normal flow patterns post-surgery.

CRT: cardiac resynchronization therapy; DCM: dilated cardiomyopathy; HCM: hypertrophic cardiomyopathy; HFpEF: heart failure with a preserved ejection fraction; LV: left ventricle; and SDI: systolic dyssynchrony index.

## Data Availability

Not applicable.

## Abbreviations

AI: artificial intelligence; CoA: coarctation of the aorta; CHD: congenital heart disease; CRT: cardiac resynchronization therapy; DCM: dilated cardiomyopathy; GLS: global longitudinal strain; HCM: hypertrophic cardiomyopathy; HF: heart failure; HFmrEF: HF with mildly reduced ejection fraction; KED: kinetic energy dissipation; LV: left ventricle; LVEF: left ventricle ejection fraction; MACEs: major adverse cardiac events; MRI: magnetic resonance imaging; SDI: systolic dyssynchrony index; RV: right ventricle; VFM: Vector Flow Mapping; VFT: vortex formation time; 2DE: two-dimensional echocardiography; and 3DE: three-dimensional echocardiography.

## References

[1] Pergola V., Cameli M., Mattesi G., Mushtaq S., D’Andrea A., Guaricci A.I., Pastore M.C., Amato F., Dellino C.M., Motta R. (2023). Multimodality Imaging in Advanced Heart Failure for Diagnosis, Management and Follow-Up: A Comprehensive Review. J. Clin. Med..

[2] Pergola V., D’Andrea A., Galzerano D., Mantovani F., Rizzo M., Giannuario G.D., Khoury G., Polizzi V., Rabia G., Gimelli A. (2023). Unveiling the Hidden Chamber: Exploring the Importance of Left Atrial Function and Filling Pressure in Cardiovascular Health. J. Cardiovasc. Echogr..

[3] Marwick T.H., Shah S.J., Thomas J.D. (2019). Myocardial Strain in the Assessment of Patients with Heart Failure: A Review. JAMA Cardiol..

[4] Nagueh S.F., Appleton C.P., Gillebert T.C., Marino P.N., Oh J.K., Smiseth O.A., Waggoner A.D., Flachskampf F.A., Pellikka P.A., Evangelisa A. (2009). Recommendations for the evaluation of left ventricular diastolic function by echocardiography. Eur. J. Echocardiogr. J. Work. Gr. Echocardiogr. Eur. Soc. Cardiol..

[5] Sperlongano S., D’Andrea A., Mele D., Russo V., Pergola V., Carbone A., Ilardi F., Di Maio M., Bottino R., Giallauria F. (2021). Left Ventricular Deformation and Vortex Analysis in Heart Failure: From Ultrasound Technique to Current Clinical Application. Diagnostics.

[6] Pestelli G., Pergola V., Totaro G., Previtero M., Aruta P., Cecchetto A., Fiorencis A., Palermo C., Iliceto S., Mele D. (2022). Value of Left Ventricular Indexed Ejection Time to Characterize the Severity of Aortic Stenosis. J. Clin. Med..

[7] Lang R.M., Badano L.P., Tsang W., Adams D.H., Agricola E., Buck T., Faletra F.F., Franke A., Hung J., de Isla L.P. (2012). EAE/ASE recommendations for image acquisition and display using three-dimensional echocardiography. Eur. Heart J. Cardiovasc. Imaging.

[8] Marsan N.A., Bleeker G.B., Ypenburg C., Ghio S., van de Veire N.R., Holman E.R., van der Wall E.E., Tavazzi L., Schalij M.J., Bax J.J. (2008). Real-time three-dimensional echocardiography permits quantification of left ventricular mechanical dyssynchrony and predicts acute response to cardiac resynchronization therapy. J. Cardiovasc. Electrophysiol..

[9] Soliman O.I.I., Geleijnse M.L., Theuns D.A.M.J., van Dalen B.M., Vletter W.B., Jordaens L.J., Metawei A.K., Al-Amin A.M., ten Cate F.J. (2009). Usefulness of left ventricular systolic dyssynchrony by real-time three-dimensional echocardiography to predict long-term response to cardiac resynchronization therapy. Am. J. Cardiol..

[10] Becker M., Hoffmann R., Schmitz F., Hundemer A., Kühl H., Schauerte P., Kelm M., Franke A. (2007). Relation of optimal lead positioning as defined by three-dimensional echocardiography to long-term benefit of cardiac resynchronization. Am. J. Cardiol..

[11] Lang R.M., Badano L.P., Mor-Avi V., Afilalo J., Armstrong A., Ernande L., Flachskampf F.A., Foster E., Goldstein S.A., Kuznetsova T. (2015). Recommendations for cardiac chamber quantification by echocardiography in adults: An update from the American Society of Echocardiography and the European Association of Cardiovascular Imaging. J. Am. Soc. Echocardiogr. Off. Publ. Am. Soc. Echocardiogr..

[12] Yu Z.-X., Yang W., Yin W.-S., Peng K.X., Pan Y.L., Chen W.W., Du B.B., He Y.Q., Yang P. (2022). Clinical utility of left atrial strain in predicting atrial fibrillation recurrence after catheter ablation: An up-to-date review. World J. Clin. Cases.

[13] Martini L., Lisi M., Pastore M.C., Righini F.M., Rubboli A., Henein M.Y., Cameli M. (2024). The Role of Speckle Tracking Echocardiography in the Evaluation of Advanced-Heart-Failure Patients. J. Clin. Med..

[14] Brady B., King G., Murphy R.T., Walsh D. (2023). Myocardial strain: A clinical review. Ir. J. Med. Sci..

[15] Voigt J.U., Cvijic M. (2019). 2- and 3-Dimensional Myocardial Strain in Cardiac Health and Disease. JACC Cardiovasc. Imaging.

[16] Stokke T.M., Hasselberg N.E., Smedsrud M.K., Sarvari S.I., Haugaa K.H., Smiseth O.A., Edvardsen T., Remme E.W. (2017). Geometry as a Confounder When Assessing Ventricular Systolic Function: Comparison Between Ejection Fraction and Strain. J. Am. Coll. Cardiol..

[17] Gorgiladze N., Shavdia M., Gaprindashvili T., Gogua E., Gachechiladze L., Gujabidze M., Pagava Z. (2024). Detection of Cardiotoxicity Using Right Ventricular Free Wall Longitudinal Strain in Low Cardiovascular Risk Breast Cancer Patients Receiving Low-Dose Anthracycline Treatment. Cureus.

[18] Liu R., Xu L.A., Zhao Z., Han R. (2024). Application of two-dimensional speckle-tracking echocardiography in radiotherapy-related cardiac systolic dysfunction and analysis of its risk factors: A prospective cohort study. BMC Cardiovasc. Disord..

[19] Lyon A.R., López-Fernández T., Couch L.S., Asteggiano R., Aznar M.C., Bergler-Klein J., Boriani G., Cardinale D., Cordoba R., Cosyns B. (2022). 2022 ESC Guidelines on cardio-oncology developed in collaboration with the European Hematology Association (EHA), the European Society for Therapeutic Radiology and Oncology (ESTRO) and the International Cardio-Oncology Society (IC-OS): Developed by the task force on cardio-oncology of the European Society of Cardiology (ESC). Eur. Heart J..

[20] Oikonomou E.K., Kokkinidis D.G., Kampaktsis P.N., Amir E.A., Marwick T.H., Gupta D., Thavendiranathan P. (2019). Assessment of Prognostic Value of Left Ventricular Global Longitudinal Strain for Early Prediction of Chemotherapy-Induced Cardiotoxicity: A Systematic Review and Meta-analysis. JAMA Cardiol..

[21] Muckiene G., Vaitiekus D., Zaliaduonyte D., Zabiela V., Verseckaite-Costa R., Vaiciuliene D., Juozaityte E. (2023). Prognostic Impact of Global Longitudinal Strain and NT-proBNP on Early Development of Cardiotoxicity in Breast Cancer Patients Treated with Anthracycline-Based Chemotherapy. Medicina.

[22] Zhou F., Yuan H., Sun J., Ran H., Pan H., Wu P., Yang Q. (2024). Two-dimensional speckle tracking imaging cardiac motion-based quantitative evaluation of global longitudinal strain among patients with coronary Heart Disease and functions of left ventricular ischemic myocardial segment. Int. J. Cardiovasc. Imaging..

[23] Yu Z., Pan H., Cheng Z., Lu K., Hu H. (2022). Evaluation of Left Ventricular Systolic Function in Patients with Coronary Microvascular Dysfunction by Three-Dimensional Speckle-Tracking Imaging. Braz. J. Cardiovasc. Surg..

[24] Yehia A., Zaki A., Sadaka M., Azeem A.M.A.E. (2024). Incremental prognostic value of speckle tracking echocardiography and early follow-up echo assessment in predicting left ventricular recovery after reperfusion for ST-segment elevation myocardial infarction (STEMI). Echocardiography.

[25] Chang W.-T., Lin C.H., Hong C.-S., Liao C.T., Liu Y.W., Chen Z.C., Shih J.Y. (2021). The predictive value of global longitudinal strain in patients with heart failure mid-range ejection fraction. J. Cardiol..

[26] Ashish K., Faisaluddin M., Bandyopadhyay D., Hajra A., Herzog E. (2019). Prognostic value of global longitudinal strain in heart failure subjects: A recent prototype. Int. J. Cardiol. Heart Vasc..

[27] Di Salvo G., Pacileo G., Limongelli G., Baldini L., Rea A., Verrengia M., D’Andrea A., Russo M.G., Calabrò R. (2010). Non sustained ventricular tachycardia in hypertrophic cardiomyopathy and new ultrasonic derived parameters. J. Am. Soc. Echocardiogr. Off. Publ. Am. Soc. Echocardiogr..

[28] Tower-Rader A., Betancor J., Popovic Z.B., Sato K., Thamilarasan M., Smedira N.G., Lever H.M., Desai M.Y. (2017). Incremental Prognostic Utility of Left Ventricular Global Longitudinal Strain in Hypertrophic Obstructive Cardiomyopathy Patients and Preserved Left Ventricular Ejection Fraction. J. Am. Heart Assoc..

[29] Zhuang H., Yang K., Zhao S., Wu J., Xu N., Zhang L., Qi X., Zhang M., Song L., Pang K. (2024). Incremental value of myocardial global longitudinal strain in predicting major adverse cardiac events among patients with hypertrophic cardiomyopathy. Echocardiography.

[30] Chen P., Aurich M., Greiner S., Maliandi G., Müller-Hennessen M., Giannitsis E., Meder B., Frey N., Pleger S., Mereles D. (2024). Prognostic relevance of global work index and global constructive work in patients with non-ischemic dilated cardiomyopathy. Int. J. Cardiovasc. Imaging.

[31] Dorbala S., Ando Y., Bokhari S., Dispenzieri A., Falk R.H., Ferrari V.A., Fontana M., Gheysens O., Gillmore J.D., Glaudemans A.W.J.M. (2019). ASNC/AHA/ASE/EANM/HFSA/ISA/SCMR/SNMMI Expert Consensus Recommendations for Multimodality Imaging in Cardiac Amyloidosis: Part 2 of 2-Diagnostic Criteria and Appropriate Utilization. J. Card. Fail..

[32] Garcia-Pavia P., Rapezzi C., Adler Y., Arad M., Basso C., Brucato A., Burazor I., Caforio A.L.P., Damy T., Eriksson U. (2021). Diagnosis and treatment of cardiac amyloidosis: A position statement of the ESC Working Group on Myocardial and Pericardial Diseases. Eur. Heart J..

[33] Behar J.M., Claridge S., Jackson T., Sieniewicz B., Porter B., Webb J., Rajani R., Kapetanakis S., Carr-White G., Rinaldi C.A. (2017). The role of multi modality imaging in selecting patients and guiding lead placement for the delivery of cardiac resynchronization therapy. Expert Rev. Cardiovasc. Ther..

[34] Antoniou N., Kalaitzoglou M., Tsigkriki L., Baroutidou A., Tsaousidis A., Koulaouzidis G., Giannakoulas G., Charisopoulou D. (2024). Speckle Tracking Echocardiography in Patients with Non-Ischemic Dilated Cardiomyopathy Who Undergo Cardiac Resynchronization Therapy: A Narrative Review. Diagnostics.

[35] Glikson M., Nielsen J.C., Kronborg M.B., Michowitz Y., Auricchio A., Barbash I.M., Barrabés J.A., Boriani G., Braunschweig F., Brignole M. (2021). 2021 ESC Guidelines on cardiac pacing and cardiac resynchronization therapy: Developed by the Task Force on cardiac pacing and cardiac resynchronization therapy of the European Society of Cardiology (ESC) with the special contribution of the European Hear. Eur. Heart J..

[36] Smiseth O.A., Torp H., Opdahl A., Haugaa K.H., Urheim S. (2016). Myocardial strain imaging: How useful is it in clinical decision making?. Eur. Heart J..

[37] Khan F.Z., Virdee M.S., Palmer C.R., Pugh P.J., O’Halloran D., Elsik M., Read P.A., Begley D., Fynn S.P., Dutka D.P. (2012). Targeted left ventricular lead placement to guide cardiac resynchronization therapy: The TARGET study: A randomized, controlled trial. J. Am. Coll. Cardiol..

[38] Alahdab F., Ahmed A.I., Nayfeh M., Han Y., Abdelkarim O., Alfawara M.S., Little S.H., Reardon M.J., Faza N.N., Goel S.S. (2024). Myocardial Blood Flow Reserve, Microvascular Coronary Health, and Myocardial Remodeling in Patients with Aortic Stenosis. J. Am. Heart Assoc..

[39] Le T.-T., Huang W., Singh G.K., Toh D.F., Ewe S.H., Tang H.C., Loo G., Bryant J.A., Ang B., Tay E.L. (2021). Echocardiographic Global Longitudinal Strain Is Associated with Myocardial Fibrosis and Predicts Outcomes in Aortic Stenosis. Front. Cardiovasc. Med..

[40] Daios S., Anastasiou V., Bazmpani M.-A., Angelopoulou S.M., Karamitsos T., Zegkos T., Didagelos M., Savopoulos C., Ziakas A., Kamperidis V. (2024). Moving from left ventricular ejection fraction to deformation imaging in mitral valve regurgitation. Curr. Probl. Cardiol..

[41] Sonaglioni A., Fagiani V., Nicolosi G.L., Lombardo M. (2024). Echocardiographic assessment of left ventricular mechanics in individuals with mitral valve prolapse: A systematic review and meta-analysis. Int. J. Cardiovasc. Imaging.

[42] Egbe A.C., Miranda W.R., Anderson J.H., Pellikka P.A., Connolly H.M. (2022). Prognostic Value of Left Ventricular Global Longitudinal Strain in Patients With Congenital Heart Disease. Circ. Cardiovasc. Imaging.

[43] Dorobantu D.M., Amir N.H., Wadey C.A., Sharma C., Stuart A.G., Williams C.A., Pieles G.E. (2024). The Role of Speckle-Tracking Echocardiography in Predicting Mortality and Morbidity in Patients with Congenital Heart Disease: A Systematic Review and Meta-analysis. J. Am. Soc. Echocardiogr..

[44] Di Salvo G., Pergola V., Fadel B., Bulbul Z.A., Caso P. (2015). Strain Echocardiography and Myocardial Mechanics: From Basics to Clinical Applications. J. Cardiovasc. Echogr..

[45] Borrelli N., Di Salvo G., Sabatino J., Ibrahim A., Avesani M., Sirico D., Josen M., Penco M., Fraisse A., Michielon G. (2020). Serial changes in longitudinal strain are associated with outcome in children with hypoplastic left heart syndrome. Int. J. Cardiol..

[46] Festa P., Lovato L., Bianco F., Alaimo A., Angeli E., Baccano G., Barbi E., Bennati E., Bonhoeffer P., Bucciarelli V. (2024). Recommendations for cardiovascular magnetic resonance and computed tomography in congenital heart disease: A consensus paper from the CMR/CCT Working Group of the Italian Society of Pediatric Cardiology and the Italian College of Cardiac Radiology endorsed by the Italian Society of Medical and Interventional Radiology (Part II). J. Cardiovasc. Med..

[47] Di Salvo G., Pacileo G., Limongelli G., Verrengia M., Rea A., Santoro G., Gala S., Castaldi B., D’Andrea A., Caso P. (2007). Abnormal regional myocardial deformation properties and increased aortic stiffness in normotensive patients with aortic coarctation despite successful correction: An ABPM, standard echocardiography and strain rate imaging study. Clin. Sci..

[48] Egbe A.C., Miranda W.R., Ahmed M., Burchill L.J., Jain C.C., Karnakoti S., Kandlakunta S., Connolly H.M. (2024). Diagnostic and Prognostic Role of Left Ventricular Strain Imaging in Adults with Coarctation of aorta. Am. J. Cardiol..

[49] Kheradvar A., Houle H., Pedrizzetti G., Tonti G., Belcik T., Ashraf M., Lindner J.R., Gharib M., Sahn D. (2010). Echocardiographic particle image velocimetry: A novel technique for quantification of left ventricular blood vorticity pattern. J. Am. Soc. Echocardiogr. Off. Publ. Am. Soc. Echocardiogr..

[50] Mele D., Smarrazzo V., Pedrizzetti G., Capasso F., Pepe M., Severino S., Luisi G.A., Maglione M., Ferrari R. (2019). Intracardiac Flow Analysis: Techniques and Potential Clinical Applications. J. Am. Soc. Echocardiogr. Off. Publ. Am. Soc. Echocardiogr..

[51] Kim W.Y., Walker P.G., Pedersen E.M., Poulsen J.K., Oyre S., Houlind K., Yoganathan A.P. (1995). Left ventricular blood flow patterns in normal subjects: A quantitative analysis by three-dimensional magnetic resonance velocity mapping. J. Am. Coll. Cardiol..

[52] Stugaard M., Koriyama H., Katsuki K., Masuda K., Asanuma T., Takeda Y., Sakata Y., Itatani K., Nakatani S. (2015). Energy loss in the left ventricle obtained by vector flow mapping as a new quantitative measure of severity of aortic regurgitation: A combined experimental and clinical study. Eur. Heart J.—Cardiovasc. Imaging.

[53] Uejima T., Koike A., Sawada H., Aizawa T., Ohtsuki S., Tanaka M., Furukawa T., Fraser A.G. (2010). A new echocardiographic method for identifying vortex flow in the left ventricle: Numerical validation. Ultrasound Med. Biol..

[54] Fiorencis A., Pepe M., Smarrazzo V., Martini M., Severino S., Pergola V., Evangelista M., Incarnato P., Previtero M., Maglione M. (2022). Noninvasive Evaluation of Intraventricular Flow Dynamics by the HyperDoppler Technique: First Application to Normal Subjects, Athletes, and Patients with Heart Failure. J. Clin. Med..

[55] Kilner P.J., Yang G.Z., Wilkes A.J., Mohiaddin R.H., Firmin D.N., Yacoub M.H. (2000). Asymmetric redirection of flow through the heart. Nature.

[56] Rodríguez Muñoz D., Moya Mur J.L., Fernández-Golfín C., Becker Filho D.C., González Gómez A., Fernández Santos S., Lázaro Rivera C., Rincón Díaz L.M., Casas Rojo E., Zamorano Gómez J.L. (2015). Left ventricular vortices as observed by vector flow mapping: Main determinants and their relation to left ventricular filling. Echocardiography.

[57] Poh K.K., Lee L.C., Shen L., Chong E., Tan Y.L., Chai P., Yeo T.C., Wood M.J. (2012). Left ventricular fluid dynamics in heart failure: Echocardiographic measurement and utilities of vortex formation time. Eur. Heart J. Cardiovasc. Imaging.

[58] Gharib M., Rambod E., Kheradvar A., Sahn D.J., Dabiri J.O. (2006). Optimal vortex formation as an index of cardiac health. Proc. Natl. Acad. Sci. USA.

[59] Pugliese N.R., Colli A., Falcetta G., Del Punta L., Puccinelli C., Fiocco A., Petronio A.S., Taddei S., Masi S., Besola L. (2023). Flow dynamic assessment of native mitral valve, mitral valve repair and mitral valve replacement using vector flow mapping intracardiac flow dynamic in mitral valve regurgitation. Front. Cardiovasc. Med..

[60] Kheradvar A., Rickers C., Morisawa D., Kim M., Hong G.-R., Pedrizzetti G. (2019). Diagnostic and prognostic significance of cardiovascular vortex formation. J. Cardiol..

[61] Pagel P.S., Hudetz J.A. (2013). Chronic pressure-overload hypertrophy attenuates vortex formation time in patients with severe aortic stenosis and preserved left ventricular systolic function undergoing aortic valve replacement. J. Cardiothorac. Vasc. Anesth..

[62] Pagel P.S., Boettcher B.T., De Vry D.J., Freed J.K., Iqbal Z. (2016). Moderate Aortic Valvular Insufficiency Invalidates Vortex Formation Time as an Index of Left Ventricular Filling Efficiency in Patients with Severe Degenerative Calcific Aortic Stenosis Undergoing Aortic Valve Replacement. J. Cardiothorac. Vasc. Anesth..

[63] Borrelli N., Avesani M., Sabatino J., Ibrahim A., Josen M., Paredes J., Di Salvo G. (2021). Blood speckle imaging: A new echocardiographic approach to study fluid dynamics in congenital heart disease. Int. J. Cardiol. Congenit. Heart Dis..

[64] Goliasch G., Goscinska-Bis K., Caracciolo G., Nakabo A., Smolka G., Pedrizzetti G., Narula J., Sengupta P.P. (2013). CRT improves LV filling dynamics: Insights from echocardiographic particle imaging velocimetry. JACC Cardiovasc. Imaging.

[65] Mangual J.O., Kraigher-Krainer E., De Luca A., Toncelli L., Shah A., Solomon S., Galanti G., Domenichini F. (2013). Comparative numerical study on left ventricular fluid dynamics after dilated cardiomyopathy. J. Biomech..

[66] Ouyang D., He B., Ghorbani A., Yuan N., Ebinger J., Langlotz C.P., Heidenreich P.A., Harrington R.A., Liang D.H., Ashley E.A. (2020). Video-based AI for beat-to-beat assessment of cardiac function. Nature.

[67] Marchese P., Cantinotti M., Van den Eynde J., Assanta N., Franchi E., Pak V., Santoro G., Koestenberger M., Kutty S. (2021). Left ventricular vortex analysis by high-frame rate blood speckle tracking echocardiography in healthy children and in congenital heart disease. Int. J. Cardiol. Heart Vasc..

